# First Reported Case of Glucose-6-Phosphate Isomerase Deficiency in a Saudi Child With Hemolytic Anemia

**DOI:** 10.7759/cureus.94817

**Published:** 2025-10-17

**Authors:** Wejdan Alotaibi, Shady Wafa, Lina Elzubair, Ayman Abualama, Badriah G Alasmari

**Affiliations:** 1 Pediatrics, Armed Forces Hospital Southern Region, Khamis Mushait, SAU; 2 Pathology, Armed Forces Hospital Southern Region, Khamis Mushait, SAU; 3 Pediatrics, Armed Forces Hospital Southern Region, Khamis Mushayt, SAU

**Keywords:** chronic hemolytic anemia, enzymopathy, first saudi patient, glucose-6-phosphate isomerase (gpi), whole-genome sequencing

## Abstract

Glucose-6-Phosphate Isomerase (GPI) deficiency constitutes a rare autosomal recessive enzymopathy that causes hereditary nonspherocytic hemolytic anemia (HNSHA). The condition is linked to homozygous or compound heterozygous mutations in the *GPI* gene located on chromosome 19q13. This enzyme deficiency disrupts glycolysis, leading to hemolysis of red blood cells. This report documents the first Saudi patient officially diagnosed with GPI deficiency. The diagnosis was conclusively established using whole-exome sequencing (WES), which identified a homozygous pathogenic variant in the *GPI *gene.

## Introduction

Glucose-6-Phosphate Isomerase (GPI) deficiency is recognized as an uncommon etiology of hereditary nonspherocytic hemolytic anemia (HNSHA) [1]. This autosomal recessive (AR) enzymopathy arises from pathogenic mutations within the *GPI *gene located on chromosome 19q13 [1]. The GPI enzyme exhibits structural and functional duality. In its homodimeric form, consisting of two identical subunits, it performs an essential catalytic role in the glycolytic pathway (glycolysis). Specifically, GPI catalyzes the second step, the interconversion of glucose-6-phosphate to fructose-6-phosphate (2). A deficiency in this enzyme thus compromises the glycolytic cascade, which is crucial for red cell energy production, ultimately impairing erythrocyte structural integrity and resulting in hemolysis [2]. Conversely, the single-molecule, or monomeric, structure of GPI is indistinguishable from neuroleukin (NLK), a neurotrophic factor that promotes the survival and maintenance of various nerve cell types, including sensory and skeletal neurons [3]. This dual role explains why a subset of patients with GPI deficiency may present with abnormal neurological complications, such as mixed sensory and cerebellar ataxia, muscle weakness, cognitive impairment, or epilepsy [4]. Furthermore, GPI is reported to function as a tumor-secreted cytokine and an angiogenic factor, mediating endothelial cell movement [5].

Due to its rarity, GPI deficiency is frequently misdiagnosed as more prevalent causes of chronic hemolytic anemia, such as G6PD deficiency or hereditary spherocytosis. Diagnosis is typically established by quantifying enzyme activity in erythrocytes, followed by confirmatory DNA sequencing of the *GPI *gene [5]. Clinical severity varies significantly, yet most affected individuals exhibit normal growth and life expectancy. Management involves supportive care, primarily through intermittent transfusions, folic acid supplementation, and vigilance regarding precipitating factors like infections and oxidative medications [6].

In this report, we document the first case of a 6-year-old male patient from the southern region of Saudi Arabia diagnosed with glucose-6-phosphate isomerase deficiency using whole exome sequencing (WES).

## Case presentation

A 6-year-old Saudi boy was referred to our hematology clinic from family medicine for evaluation of frequent hemolytic anemia episodes requiring multiple blood transfusions. He was born preterm at 34 weeks via Caesarean section to consanguineous parents. Following birth, he was admitted to the neonatal intensive care unit for two months due to prematurity but was discharged without any reported complications. He first presented to our clinic at the age of four years and six months. His mother reported recurrent complaints of pallor and fatigue, along with episodes of hemolysis, which were typically preceded by an upper respiratory tract infection. The mother denied a history of changes in urine color, bleeding disorders, or recurrent infections.

By the age of 1.5 years, the patient had already received two blood transfusions after two hemolytic attacks without any identifiable cause. The mother also noted that the patient's older sister experienced similar, undiagnosed manifestations that improved spontaneously throughout her life. We asked the mother to follow his sister with the adult hematology clinic, as she was an adult now. On clinical examination, the patient appeared pale but was developmentally appropriate for his age. Physical assessment was unremarkable: there was no evidence of jaundice, bruising, bleeding, petechiae, or lymphadenopathy, and no palpable hepatosplenomegaly. Other systemic examinations were normal.

**Figure 1:**
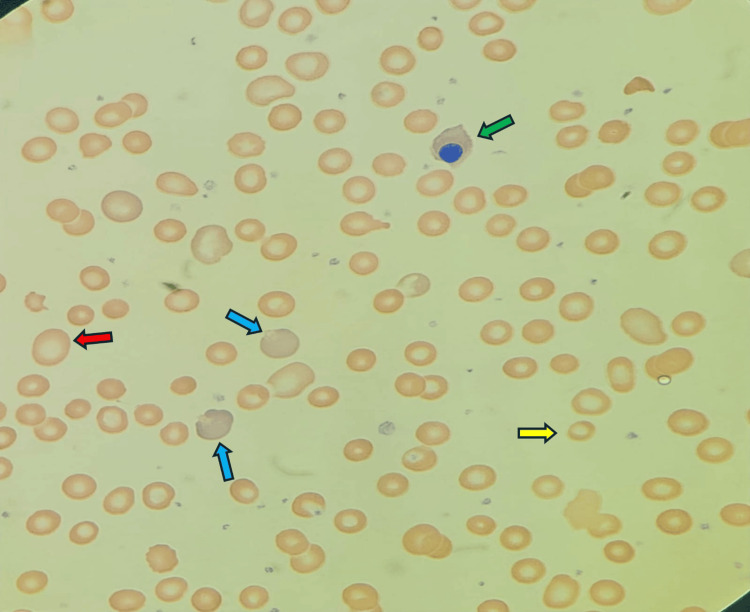
Peripheral blood smear. Peripheral blood smear showing nucleated red blood cells (green arrow), polychromatic cells (blue arrow), macrocytes (red arrow), and hypochromic microcytic red blood cells (yellow arrow).

Initial laboratory investigations indicated severe anemia with normocytic to mildly macrocytic red cell indices (MCV ≈95 fl), which ruled out iron deficiency and suggested a hemolytic process (Table 1). Both lactate dehydrogenase (LDH) and serum ferritin levels were elevated, alongside a slight elevation in total serum bilirubin (Table 1). A direct Coombs test was negative, and the glucose-6-phosphate dehydrogenase (G6PD) enzyme level was normal. The peripheral blood smear revealed normocytic, normochromic anisopoikilocytosis with numerous polychromatic cells and the presence of nucleated red blood cells (Figure 1). Following a corrective blood transfusion for severe anemia, the patient was regularly monitored in our clinic for rare causes of hemolytic anemia.

**Table 1:** Key laboratory findings of the patient. Critical abnormal values indicative of severe hemolytic anemia and iron overload are highlighted in bold WBC=White Blood Cell count, RBC=Red Blood Cell count, HCT=Hematocrit, MCV=Mean Corpuscular Volume, RDW=Red Cell Distribution Width, PLT=Platelet count, LDH=Lactate Dehydrogenase, HPLC=High-Performance Liquid Chromatography, Hb=hemoglobin

Description	Result	Unit	Reference range
WBC	6.00	10^9^/L	4.5-13.5
RBC	2.09	10^12^/L	4.1-5.3
Hb	5	g/dl	10.9-15
HCT	19.80	%	31-41
MCV	94.60	fl	73-98
RDW	14.90	%	
PLT	495	10^9^/L	150-450
Reticulocyte	10	%	0.5-2.5
LDH	500	Mmol/L	155-290
Total bilirubin	43	μmol/L	< 34
Direct bilirubin	7	μmol/L	1.7-8.6
Ferritin	2000	μg/l	10.3-55.8
HPLC (Hemoglobin Electrophoresis)			
Hb A2	1.6	%	1-3.5
Hb A	97.6	%	96.5-99
Hb S	0	%	0
Hb F	0.8	%	2

At five years of age, he experienced another acute attack of severe anemia and hemolysis requiring hospital admission and blood transfusion. At the time of this admission, Whole Exome Sequencing (WES) was performed. The results were positive, identifying the specific homozygous pathogenic variant in the *GPI *gene: c.1532G > A p.(Arg511His). This finding was consistent with the diagnosis of autosomal recessive congenital nonspherocytic hemolytic anemia type 4. Following the confirmed molecular diagnosis, the patient’s long-term management was initiated, consisting of intermittent transfusions and folic acid supplementation. Genetic counseling was provided to the family. During follow-up, he has remained clinically stable without any signs of neurological involvement.

## Discussion

GPI deficiency is an uncommon enzymopathy characterized by variable clinical severity; approximately 90 patients have been documented globally across various ethnic populations (6). While some affected individuals maintain a state of compensated anemia, others progress to severe, transfusion-dependent disease. Although neurological manifestations have been reported in a minority of cases, these symptoms were absent in our patient. This case highlights the diagnostic challenges associated with GPI deficiency, particularly as the initial clinical picture frequently overlaps with more common hemolytic disorders. While specialized enzyme activity assays are beneficial, their accessibility is often limited, especially in resource-constrained settings. Conversely, the implementation of next-generation sequencing (NGS) enables a rapid and conclusive molecular diagnosis, as demonstrated by the findings presented here.

A previously published study described a 4-year-and-6-month-old boy who presented with similar hematological manifestations but lacked associated neurological symptoms [7]. That patient was also diagnosed via whole-exome sequencing, which identified the *GPI *gene variant c.1040G > A p.Arg347His [7], a different molecular lesion from the one observed in our case. Furthermore, other research has linked *GPI *mutations to neurological sequelae, proposing that the underlying mechanism involves incorrect protein folding that destroys both the catalytic (GPI) and neurotrophic (NLK) activities of the enzyme [8]. GPI deficiency can also present as a life-threatening condition in utero. One report described a family with a history of an affected 5-year-old daughter and recurrent early miscarriages; during a subsequent pregnancy, fetal anemia was detected at 26 weeks of gestation via increased Middle Cerebral Artery Peak Systolic Velocity (MCA-PSV) >1.5 Multiples of the Median, necessitating intrauterine blood transfusion (IUT) [9].

The management of GPI deficiency is primarily supportive, focusing on transfusion therapy when necessary, folic acid supplementation, and vigilant monitoring for potential iron overload. Although splenectomy has been attempted in certain individuals, the therapeutic outcomes remain inconsistent. Given the autosomal recessive inheritance pattern and the potential for severe outcomes, genetic counseling is a vital component of patient care, particularly in populations where consanguinity rates are high.

## Conclusions

This is the first reported Saudi case of GPI deficiency confirmed through molecular testing. This report underscores the importance of thoroughly considering rare red cell enzymopathies in the differential diagnosis of patients with otherwise unexplained hemolytic anemia. Early utilization of genetic testing facilitates precise diagnosis, allows for appropriate and timely management, and enables comprehensive family counselling.

## References

[1] Manco L, Bento C, Victor BL (2016). Hereditary nonspherocytic hemolytic anemia caused by red cell glucose-6-phosphate isomerase (GPI) deficiency in two Portuguese patients: clinical features and molecular study. Blood Cells Mol Dis.

[2] Warang P, Kedar P, Ghosh K, Colah RB (2012). Hereditary non-spherocytic hemolytic anemia and severe glucose phosphate isomerase deficiency in an Indian patient homozygous for the L487F mutation in the human GPI gene. Int J Hematol.

[3] Burger NC, van Wijk R, Bresters D, Schell EA (2019). A novel mutation of glucose phosphate isomerase (GPI) causing severe neonatal anemia due to gpi deficiency. J Pediatr Hematol Oncol.

[4] Schröter W, Eber SW, Bardosi A, Gahr M, Gabriel M, Sitzmann FC (1985). Generalised glucosephosphate isomerase (GPI) deficiency causing haemolytic anaemia, neuromuscular symptoms and impairment of granulocytic function: a new syndrome due to a new stable GPI variant with diminished specific activity (GPI Homburg). Eur J Pediatr.

[5] Kedar PS, Gupta V, Dongerdiye R, Chiddarwar A, Warang P, Madkaikar MR (2019). Molecular diagnosis of unexplained haemolytic anaemia using targeted next-generation sequencing panel revealed (p.Ala337Thr) novel mutation in GPI gene in two Indian patients. J Clin Pathol.

[6] Kugler W, Lakomek M (2000). Glucose-6-phosphate isomerase deficiency. Baillieres Best Pract Res Clin Haematol.

[7] Dhankar M, Mandal P, Singh R (2025). Unusual cause of hemolytic anaemia: glucose phosphate isomerase deficiency. Pediatr Hematol Oncol J.

[8] Kugler W, Breme K, Laspe P (1998). Molecular basis of neurological dysfunction coupled with haemolytic anaemia in human glucose-6-phosphate isomerase (GPI) deficiency. Hum Genet.

[9] Adama van Scheltema PN, Zhang A, Ball LM, Steggerda SJ, van Wijk R, Fransen van de Putte DE, van Kamp IL (2015). Successful treatment of fetal hemolytic disease due to glucose phosphate isomerase deficiency (GPI) using repeated intrauterine transfusions: a case report. Clin Case Rep.

